# Psychological Distress in Older Adults Living in Puerto Rico during COVID-19

**DOI:** 10.1007/s10823-025-09539-8

**Published:** 2025-06-24

**Authors:** David Camacho, Matthew R. Morgan, Julia Vazquez, Jerad H. Moxley, Denise Burnette

**Affiliations:** 1 Department of Disability and Human Development, University of Illinois, 1640 Roosevelt Road #712, Chicago, IL 60608 USA; 2 School of Social Work, University of North Carolina at Chapel Hill, 325 Pittsboro Street, Chapel Hill, NC 27599 USA; 3 School of Social Work, Virginia Commonwealth University, 1000 Floyd Avenue, Richmond, VA 23284 USA; 4 School of Social Work, University of Maryland, 525 Redwood Street, Baltimore, MD 21201 USA; 5 Weill Cornell Medicine Center on Aging and Behavioral Research, Division of Geriatrics and Palliative Medicine at Weill Cornell Medicine, 420 East 70th Street 3rd Floor, Suite B, New York, NY 10021 USA

## Abstract

Few studies have addressed the impact of COVID-19 on mental health in low resource settings. This study examines the association of COVID-19-related stressors with psychological distress in older adults in Puerto Rico. Data are from a 2021 Knowledge, Attitudes and Practices survey about COVID-19 with adults aged 60 + in Puerto Rico (*n* = 213). We used the Self-Reporting Questionnaire-20 (SRQ-20) to assess distress. Stressors included COVID-19 diagnosis, hospitalization, or death of someone close, treatment delays, and loneliness. We used negative binomial regression with a log linear link function to model the effects of demographics and pandemic-related social and health stressors on distress. Almost one-third (31%) of participants reported clinically significant distress; 44.3% reported a loved one diagnosed with COVID-19, 32.4% had lost someone close, 25.8% reported treatment delays, and 39.4% experienced loneliness. A COVID-19 diagnosis of someone close without hospitalization [Adjusted Incidence Rate Ratio (AIRR) = 1.55; 95% CI 1.08, 2.22] and loneliness [AIRR = 1.20; 95% CI 1.09, 1.32] were associated with greater SRQ-20 scores. We consider the nexus of cultural and contextual factors (e.g., outmigration, under-resourced healthcare system, fatalism etc.) that are likely to influence short- and longer-term effects of COVID-19-related distress among older adults in Puerto Rico. Interdisciplinary collaborations are needed to enhance Covid-19-related support and to implement culturally appropriate and contextually feasible evidence-based interventions that will reduce high rates of mental health challenges and prevent their long-term effects.

## Introduction and Background

More than 770 million people have contracted COVID-19, of whom nearly seven million have died (World Health Organization, 2023). Compared to younger persons, older adults are more likely to be hospitalized and to die of COVID-19 complications (Mueller et al., 2020). To control the pandemic, public health authorities implemented compulsory lockdowns and personal safety guidelines (Papadopoulos et al., 2020). These restrictions have seriously limited in-person socialization (Saladino et al., 2020), raising risks of social isolation and loneliness and interrupting medical care for existing health conditions that disproportionately affect older adults (Nouri et al., 2020).

The immediate and long-term impact of COVID-19 on mental health is a public health concern worldwide (see Mendez-Lopez et al., 2022). Depressive and anxiety symptoms may indicate the presence of acute or chronic mood disorders that worsen quality of life (Canuto et al., 2018; Sivertsen et al., 2015) and cognitive functioning (Gulpers et al., 2019; Ruthirakuhan et al., 2019) and increase medical costs (Chisholm et al., 2016) and premature mortality (Wu et al., 2020).

Having a family member or friend who has been diagnosed (Burke et al., 2020; Gallagher et al., 2020), hospitalized (Burke et al., 2020; Gallagher et al., 2020; Khubchandani et al., 2022; Paccagnella & Pongiglione, 2022), or who has died from COVID-19 (Breen et al., 2021; Burke et al., 2020; Joaquim et al., 2021; Khubchandani et al., 2022; Paccagnella & Pongiglione, 2022) and COVID-19 related delays in medical care (Bui et al., 2021; Jalan et al., 2022; Leach et al., 2021) can contribute to increased symptoms of depression and anxiety. Loneliness refers to the distressing feeling that occurs when individuals perceive their social needs are not met by the quantity and particularly the quality of their social relationships (Hawkley & Cacioppo, 2010; Perlman & Peplau, 1981) and is also associated depressive and anxiety symptoms (Dziedzic et al., 2021; Killgore et al., 2020).

Drawing on this body of research, we hypothesize that COVID-19 related events (i.e., infection, hospitalization, and death of loved ones) will be associated with higher levels of depressive and anxiety symptoms (Beck, 1967; Francis et al., 2012). Contextual factors such as healthcare resources (Correa et al., 2020), cultural perspectives, including perceived causes and idioms of distress (Kleinman, 2020); and aging processes (Carstensen, 1992) may also influence older adults’ experience and expression of pandemic-related symptoms of depression and anxiety.

Before the pandemic, Prina and colleagues (2019) found that 16.5% of Puerto Ricans aged 65 years and older who participated in the 10/66 cohort study reported clinically significant depressive symptom based on the *Classification of Diseases*,* Tenth Revision* (ICD-10) and EURO-D. In a study with ​​45 adults in Puerto Rico (60–86 years), Taylor and Irizarry-Robles (2015) found that more 68% experienced depressive symptoms based on the Zung Self Rating Depression Scale. In another study of 491 community dwelling older adults in Puerto Rico, 46% reported that they felt unhappy or depressed during the previous month (Oliver-Vazquez et al., 1999). Wu et al. (2020) found that approximately 5% of all participants from six Latin American countries (including Puerto Rico) from the 10/66 cohort study met clinically significant anxiety symptoms based on the Geriatric Mental State (GMS) examination. The authors did not report country-specific prevalence. More recently, Cameron-Maldonado et al. (2023) used data from an online survey with 1,945 adults aged 18 years and older during the COVID-19 pandemic (December 2020 - February 2021) to assess self-reported provider-diagnosed depression and anxiety. Among participants 50 years and older, 17% reported receiving a diagnosis of depression and 19% reported a diagnosis of anxiety.

Beyond these studies, COVID-19 related depression and anxiety in older adults in Puerto Rico is very limited. Contextual, cultural, and age-related factors are expected to contribute to variability in COVID-19-related mental health challenges in this population. For example, 23.5% of the archipelago’s 3.2 million people are over the age of 65 years (US Census, 2022) and more than 40% of older adults live under the federal poverty line (U.S. Census Bureau, 2021). They are disproportionately affected by chronic health conditions (Garcia, Garcia & Ailshire, 2018), yet the healthcare infrastructure is poorly equipped to address their needs. The majority of the 60 hospitals are in urban areas (Health Resources & Services Administration, 2023) and there are shortages of physicians, specialized medical care services, and medical supplies (see Beaton- Comulada et al., 2022; Perreira et al., 2017). Further, in the years preceding the pandemic, Puerto Rico endured hurricanes and earthquakes that damaged hospital structures and interrupted electricity, potable water supplies, and communication systems.

Common perspectives of mental health among Puerto Rican adults may negatively impact identification and management of psychological conditions. Older adults may use cultural idioms to conceptualize and describe symptoms of depression and anxiety, e.g., *ataque de nervios* (“attack of nerves”; Guarnaccia et al., 2003), and they may be unwilling to acknowledge symptoms to avoid being labeled ‘crazy’ (Negroni et al., 2020). Somatization of symptoms as physical complaints is also common (Guarnaccia et al., 2003), and mental health treatment is limited (Negroni et al., 2020). Further, common Latino beliefs and mourning practices can provide comfort and support for the bereaved and mitigate related psychological distress (see Falzarano et al., 2022). For example, fatalism (the conviction that God controls the future), and participating in wakes (may include prayer, food, offerings) can serve as a source of comfort for bereaved older adults.

As of September 2023 in Puerto Rico, there were more than 1.25 million confirmed cases of COVID-19, and close to 6,000 COVID-19 related deaths (World Health Organization, 2023). COVID-19 continues to be a threat to older adults in Puerto Rico, yet very few studies have investigated mental health symptoms in this population during the COVID-19 pandemic. We hypothesize that common COVID-19 related experiences (i.e., infection, hospitalization, and/or death of a person close to the older adult, participant’s delays in treatment, feelings of loneliness) will be associated with higher levels of psychological distress. Finally, we interpret our findings based on Puerto Rico’s contextual and cultural realities and potential long-term implications for health and well-being.

## Methods

### Data Source

Data were from a Knowledge, Attitudes and Practices during COVID-19 study of older adults in Puerto Rico. Surveys were conducted from January through December, 2021 via telephone and face-to-face interviews with a nonprobability sample of adults aged 60+ (*N* = 213) from all six geographic regions. Data on sociodemographic characteristics, social relationships, physical and mental health, elder mistreatment, and personal/interpersonal experiences of infection and loss due to COVID-19 were collected in Spanish. Inclusion criteria were aged 60 years and over, native of Puerto Rico and living there during the pandemic, and capable of providing informed consent. The interviewers were social work researchers who have extensive experience with older adults, mental health assessment and treatment, and Puerto Rican and diverse Latinx communities. Original study interview was composed in English. Puerto Rican team members translated the English version to Spanish. The original Spanish translation version was piloted with ten older adults in Puerto Rico. Pilot data led to several minor revisions to better reflect the cultural context.

With support from community agencies and the archdiocese of Puerto Rico, we recruited participants from community and senior centers, social service agencies, religious organizations, and social spaces such as town plazas and private porches. The study was approved by the (Virginia Commonwealth University) Institutional Review Board.

### Measures

#### Outcome

Psychological Distress was assessed using the Self Reporting Questionnaire (SRQ-20; Harding et al., 1980). The SRQ-20 is widely validated for identifying common mental health disorders in low- and middle-income countries (LMIC) (e.g., Ali et al., 2016) including those in Latin America (e.g., Chile: Araya et al., 1992). The SRQ-20 also aligns well with cultural conceptualizations of depression and anxiety (e.g., somatization; Guarnaccia et al., 2003). The instrument assesses symptoms of depression (e.g., feeling unhappy), anxiety (e.g., feeling nervous, tense, or worried) and somatic symptoms (e.g., headaches, poor digestion, sleeping difficulty). Possible responses are yes = 1 and no = 0 (theoretical range: 0 to 20) with higher scores reflecting greater levels of psychological distress.

### Primary Predictors

Participants were asked two questions using the same response categories: “Have you or someone close to you been diagnosed with COVID-19?” and “Have you or someone close to you (a friend or relative) been hospitalized with COVID-19?” (self only = 1, someone close only = 2, or both self and someone close = 3 and neither = 4). Three participants reported being diagnosed and one was hospitalized with COVID-19. We combined responses to the 2 questions as follows: no diagnosis or hospitalization of loved one due to COVID-19 = 1 (reference); someone close to me diagnosed with COVID-19 but not hospitalized = 2 and someone close to me diagnosed and hospitalized due to COVID-19 = 3.

Death of someone close was measured by the question, “Has someone close to you died during the COVID-19 pandemic, that is since March 2020?” (yes = 1, no = 0).

Delays in treatment were assessed by asking: “Since the COVID-19 pandemic began, have you needed medical or dental care or any medications that you had to delay or have not been able to receive?” (yes = 1, no = 0).

We measured loneliness with the Three-Item Loneliness Scale (TILS), which asked participants how often they felt a lack of companionship, left out, and isolated from others. Possible responses were: hardly ever = 1, some of the time = 2 and often = 3. Theoretical range was 3–9, with higher scores indicating higher levels of loneliness (Hughes et al., 2004).

### Covariates

We measured several health and social factors that are commonly associated with depressive and anxiety symptoms. Poorer self-rated health (Östberg & Nordin, 2022) and living alone (Teo et al., 2013; Wu et al., 2022) are positively associated with greater depressive and anxiety symptoms. Self-rated health was assessed by the question “In general, would you say that your health…” excellent = 1; very good = 2; good = 3; fair = 4, and poor = 5) (Zajacova & Dowd, 2011). Living arrangement was coded as living alone = 1; living with others = 0).

The impact of mandatory social distancing on feelings of loneliness, depression, and anxiety were a major pandemic-related concern for older adults (Benke et al., 2020; Hwang et al., 2020) We thus controlled for preventive practices with a 12-item index of preventive behaviors drawn from CDC and WHO recommendations. Examples include: social distancing (e.g., left your home to visit friends or family, avoided shaking hands, hugging, or kissing), sanitary practices (e.g., washing hands after being in public, avoiding touching face, wearing mask), and keeping up to date with recommendations (0 = often or always, 1 = sometimes; 2 = rarely or never). Negatively weighted items were reverse coded to create a sum score ranging from 0 to 24 where higher scores indicated stronger adherence to recommended preventive practices.

Sociodemographic factors associated with symptoms of depression and anxiety include age (Bryant et al., 2008; Mitchell & Subramaniam, 2005), birth sex (Farhane-Medina et al., 2022; Labaka, Goñi-Balentziaga, Lebeña & Pérez-Tejada, 2018), marital status (Pei et al., 2019; Yan et al., 2011), and educational attainment (Chang-Quan et al., 2010; Hovenkamp-Hermelink et al., 2021). Our final model adjusted for age (in years), birth sex (female = 0, male = 1), marital status (married or living as married = 1, never married = 2, divorced = 3, and widowed = 4), and educational attainment (0 = less than high school, 1 = high school, 2 = more than high school).

### Analytic Strategy

Our main aim was to examine the effects of COVID-19 experiences (COVID-19 diagnosis/hospitalization and/or death of someone close, treatment delays, and loneliness) on SRQ-20 scores. Table 1 presents descriptive and bivariate data on predictor variables and SRQ-20 scores.

**Table 1:** Descriptive statistics (*n* = 213)

	Descriptive statistics	Bivariate relationship with SRQ Score
**Psychological Distress** Score ≥ 8	5.63(5.01) 31.5%	N/A
**Common Covid-19 Challenges**		
**Dx and Hospitalization** No dx, No Hospitalization Yes dx, No Hospitalization Yes dx, Yes Hospitalization	55.8% 29.7% 14.6%	Reference ***r***_s_**(211) = 0.282. *p*<.001** *r*_s_ (211) = 0.077, *p* =.264
**Death for Any Reason** No Yes	67.6% 32.4%	Reference ***r***_***s***_**(211) =** 0.064. *p* =.355
**Delays in Care** No Yes	74.2% 25.8%	Reference ***r***_s>_**(211) = 0.209,**, ***p*=.002**
**Loneliness**	5.1(1.78)	***r***_s_**(211) = 0.474**,***p*<.001**
**Age**	71.9(8.72)	*r*_*s*_(211) = − 0.122 *p* =.079
**Sex** Male Female	44.1% 55.9%	Reference ***r***_s_**(211) = 0.156**, ***p*=.022**
**Marital Status** Married/Living with Partner Never Married Divorced Widowed	21.8% 17.3% 32.9% 28%	Reference *r*_*s*_(211) = − 0.65, *p* =.346 *r*_*s*_(211)= − 0.125, *p* =.068 *r*_*s*_(211)= 0.077, *p* =.262
**Education** More than High School High School or equivalent Less than High School	30% 28.3% 41.6%	Reference *r*_*s*_(211) = 0.029, *p* =.678 *r*_*s*_(211)= − 0.071, *p* =.308
**Living Alone** No Yes	36.6% 63.4%	Reference *r*_*s*_(211) = − 0.009, *p* =.897
**Self-Rated Health**	3.09(1.07)	***r***_s_**(211) = 0.555**,***p*** **<.001**
**Practice and Behavior Index**	20.81 (3.06)	*r*_*s*_(211) = 0.090, *p* =.192

Table 2 presents a multiple regression model with all covariate and fixed classification factors. Data points were missing for 8% of original data. We treated missing data with multiple imputation using fully conditional specification chained equation methods, with 20 imputations, using predictive mean matching for models for scale variables (Rubin, 2018). We conducted a robustness check using listwise deletion. The main inferences were unchanged. We fitted a negative binomial model with a log link function (Lindén & Mäntyniemi, 2011) as exploratory Poisson regression models indicated overdispersion with (θ > 2.7). We used SPSS version 27 for all analyses.

**Table 2:** Negative binomial regression analyses of COVID-19 experiences and SRQ-20 scores, pooled *n* = 213

	Incident Rate Ratio (95% Confidence Interval) *P*-Value
	SRQ-20 Score
Common Covid-19 Challenges	
**Dx and Hospitalization** No dx, No Hospitalization Yes dx, No Hospitalization Yes dx, Yes Hospitalization	Reference **1.55[1.08**,** 2.22] 0.018** 1.55[0.97, 2.48] 0.067
**Death for Any Reason** No Yes	Reference 1.09[0.77, 1.54] 0.636
**Delays in Care** No Yes	Reference 1.19[0.83, 1.72] 0.337
**Loneliness**	**1.20[1.09**,** 1.32] < 0.001**
**Age**	1.00[0.98, 1.01] 0.582
**Sex** Male Female	Reference 1.32[0.92, 1.89]
**Marital Status** Married/Living with Partner Never Married Divorced Widowed	Reference 0.61[0.34, 1.12] 0.112 **0.57[0.33**,** 0.99]0.047** 0.68[0.38, 1.24] 0.211
**Education** Less than High School High School or equivalent More than High School	0.96[0.65, 1.41] 0.89[0.59, 1.34] Reference
**Living Alone** No Yes	Reference 1.24[0.81, 1.91] 0.314
**Self-Rated Health**	**1.42[1.22**,** 1.65] < 0.001**
**Practice and Behavior Index**	1.00[0.94, 1.05] 0.864

## Results

### Descriptive Statistics

Table 1 shows descriptive information on our full imputed sample. The mean SRQ-20 was 5.67 (SD = 5.01); almost one-third (31.5%) of respondents met the pre-established clinical threshold of 8 indicative of clinically significant psychological distress (i.e., depression and/or anxiety symptoms; Harding et al., 1980).

In our sample, 44.3% of older adults reported that someone close to them was diagnosed with COVID-19, while 14.6% reported that someone close was hospitalized due to COVID-19. About one-third had experienced the death of a loved one during the pandemic, and 25.8% reported delays in care. More than 39% of participants met the threshold (score **≥** 6) for loneliness (Hughes et al., 2004). The mean age of participants was 71.9 years (SD = 8.72), 55.9% were female, and 21.8 were married or living with a partner. Almost two thirds (63.4%) were living alone. Almost 40% of participants rated their health as fair or poor. Finally, the mean score for preventive practices was 20.81 (SD = 3.06) indicating high adherence to recommended protocols. 

### Bivariate Results

Table 1 includes results of bivariate analyses (Spearman’s rho). Individuals who had someone close to them diagnosed with COVID-19 (not hospitalized), were experiencing delays in care [stats], had higher loneliness scores, and were female were positively associated with higher SRQ-20 scores. Poorer self-rated health was also positively associated with greater SRQ-20 scores.

### Multivariate Results

Table 2 presents our fully adjusted models. Compared to older adults who reported not having a loved one diagnosed or hospitalized with COVID-19, persons who had someone close diagnosed with COVID-19 but not hospitalized experienced significantly greater SRQ-20 scores. Those who had someone close to them diagnosed with COVID-19 and hospitalized also reported greater SRQ-20 scores but the differences were not statistically significant. Greater loneliness was positively associated with SRQ-20 scores, whereas neither death of someone close nor delay in care was associated with SRQ-20 scores.

## Discussion

Our findings add to the limited literature on mental health of older adults in Puerto Rico during the COVID-19 pandemic. Almost one-third of our sample reported clinically significant levels of psychological distress. After accounting for relevant demographic and health factors, having someone close to them diagnosed (but not hospitalized) with COVID-19 and loneliness were positively associated with higher psychological distress. These findings give insight into current and potentially longer-term mental health challenges of older adults in Puerto Rico.

First, there is limited research on the mental health of older adults in Puerto Rico, leading us to highlight that nearly one third of our community sample reported clinically significant psychological distress. Earlier studies have reported a wide range of estimates of depressive symptomatology or diagnosis (range: 16.5–68%; Cameron-Maldonado et al., 2023; Oliver-Vázquez et al., 1999; Prina et al., 2019; Taylor & Irizarry-Robles, 2015) and anxiety (5–19%; Cameron-Maldonado et al., 2023; Wu et al., 2020). Our findings of high rates of symptoms of depression and anxiety are important as they contribute to poor health and costly health outcomes, such as cognitive impairment (Alzheimer’s Association, 2023; Gulpers et al., 2019; Sivertsen et al., 2015). Even subthreshold diagnosable symptoms in older adults may contribute to poorer health conditions and worse functioning in later life (Biella et al., 2019).

About one-third of our sample reported that someone close to them was diagnosed with COVID-19 but not hospitalized, a finding that was associated with greater psychological distress (Burke et al., 2020; Gallagher et al., 2020). Having a loved one diagnosed with COVID-19 is not equally stressful for all. However, aging, health, and cultural factors may influence subjective perceptions, exacerbate stress and contribute to psychological distress. Family caregiving of a loved one with COVID-19, whether in-person or long-distance, may be a contributing factor (Adelman, 2014; Cagle & Munn, 2012). Face-to-face care may threaten carers’ health, especially if they are vulnerable due to advanced age or poor health (Liu et al., 2020; Shahid et al., 2020). Likewise, witnessing a loved one’s COVID-19 symptoms such as breathing difficulties may lead to feelings of worry and uncertainty, particularly if effective treatments cannot be obtained (Picardi et al., 2021). Long distance caregiving, on the other hand, may worsen stress by limiting care activities within social distancing guidelines (e.g., phone calls, advice, prayer). Lack of visual confirmation of their loved one’s progress may increase rumination and in turn their psychological distress.

Perceived vulnerability may also contribute to caregiver distress, particularly if the person with COVID-19 was an older adult (Shahid et al., 2020), had serious physical illness (e.g., cancer; Al-Quteimat & Amer, 2020), had multi-morbidity (Liu et al., 2020), was a head of household, or was an essential worker (Guasti, 2020). Owing to the importance of familism (Zayas & Palleja, 1988) in Puerto Rican culture, participants’ source of distress may well extend beyond the nuclear family system. An inability to work due to COVID-19, for example, may disrupt family wellbeing and functioning (Niles et al., 2020; Perri et al., 2020).

Counter to prior research (Burke et al., 2020; Gallagher et al., 2020; Khubchandani et al., 2022; Paccagnella & Pongiglione, 2022), having someone close hospitalized due to COVID-19 was not associated with psychological distress in our sample. This could be due to sampling, or because hospitalization was not widely available beyond urban areas in the early part of the pandemic (Perreira et al., 2017). The COVID-19 pandemic also depleted resources, such as mechanical ventilation, intensive care units, and medical staff (Cohen & van der Meulen Rodgers, 2020) and forced providers to ration resources to those with highest odds of survival (Hanna et al., 2020). Although hospitalizations for surgery, accidents, and illnesses (Titler et al., 1991) tend to heighten stress for patients and their loved ones, they may have caused even higher levels of stress as the Puerto Rican healthcare infrastructure was already strained due to shortages of commodities such as running water and electricity (Beaton-Comulada et al., 2022; Perreira et al., 2017). Safety guidelines further limited patients’ access to support (Voo et al., 2020) and family members’ ability to confirm their loved one’s condition.

Over a quarter of older adults in our study reported delays in medical care or medications. Unlike prior research, delays were not associated with psychological distress (Bui et al., 2021; Jalan et al., 2022; Leach et al., 2021). Chronic medical conditions are common in later life and require consistent follow up (Nouri et al., 2020), so the impact of delayed care may be highest for individuals with urgent health needs, e.g., those with life threatening conditions. Further, receiving care during the pandemic may have contributed to elevated stress levels. For example, telemedicine allowed for healthcare while maintaining social distancing (Nouri et al., 2020). However, the digital divide may have contributed to increases in stress as older adults are more likely than younger individuals to have trouble accessing and using online platforms.

One-third of older adults in our study reported the death of a loved one during the pandemic. Counter to prior research (Breen et al., 2021; Burke et al., 2020; Joaquim et al., 2021; Khubchandani et al., 2022; Paccagnella & Pongiglione, 2022) there was no association between such a death and SRQ-20 scores. Older adults in Puerto Rico have endured multiple exposures to natural disasters and related deaths, including the high death toll from hurricane María (Benach et al., 2019). The perspective of limits to the life course (Carstensen, 1992), and cultural understandings of death such as fatalism (Boucher, 2017) may have moderated subjective perspectives of death and subsequent psychological distress.

As noted, the COVID-19 pandemic disrupted social processes of dying and bereavement (Eisma & Tamminga, 2020). Patients often died without family present and were buried without proper cultural rituals (Cardoso et al., 2020; Meier et al., 2016). Limited opportunity for a “good death” in the times of COVID-19 will influence how the bereaved make sense of a loved one’s death and may ultimately be difficult to resolve (Albuquerque, 2021; Meier et al., 2016).

About 40% of study participants scored at or above the cutoff score of 6 on the TILS, indicating loneliness (Hughes et al., 2004). Similar to research conducted before and during the COVID-19 pandemic, we found that loneliness was associated with higher levels of psychological distress (Dziedzic et al., 2021; Killgore et al., 2020). Individuals with loneliness tend to report higher levels of stress (Hawkley & Cacioppo, 2003), which likely contribute to psychological distress (Beck, 1967). In our study, the relationship of loneliness and psychological distress remained after controlling for indicators of social isolation (e.g., Practice and Behavior Index, marital status, living alone; Wang et al., 2017). Few studies have addressed loneliness of older adults in Puerto Rico, although it is associated with multiple negative health outcomes in later life (e.g., Ong et al., 2016) and may vary across contexts (Ozawa-de Silva & Parsons, 2020).

Research that examines the impact of cultural and contextual factors on assessment, impact, and treatment of loneliness and related psychological distress merit deeper examination. Stigma and negative perceptions of loneliness (Lau & Gruen, 1992; Shiovitz-Ezra & Ayalon, 2012) and psychological distress (e.g., Negroni et al., 2020) may impact recognition and disclosure of mental health conditions. In our study we used a Spanish language validated measure of loneliness (TILS; Hughes et al., 2004). However, like previous work (“ataque de nervios”; Guarnaccia et al., 2003), linguistic and cultural expressions of loneliness should be explored with older adults in Puerto Rico (Camacho et al., 2025). As a subjective experience, it is important to consider how familism and altruism and coping strategies influence feelings of loneliness and related psychological distress. Further, loneliness it will be important to examine how Puerto Rico’s massive out migration (Figueroa-Rodríguez et al., 2012) of younger adults shapes older adults’ expectations of familial relationships and intergenerational support. Gaining greater understanding of how cultural and contextual based perceptions may moderate the relationships across loneliness, psychological distress, and other health outcomes is necessary (Camacho et al., 2024). These findings can support tailoring of mental health interventions that may be implemented in the Puerto Rican context.

Finally, we note that cultural and contextual factors not available in our data set may enhance our understanding of psychological distress and associated factors. For example, intergenerational support (e.g., emotional, financial, long-distance caregiving) may buffer stress and related psychological distress. Further, spiritual beliefs and prayer may enhance hope and facilitate coping to reduce stress and its negative impact on health. Other important social (e.g., gender roles, caregiving duties) and socioeconomic resources (e.g., proximity of healthcare facilities, wealth, digital resources and skills) should be considered in future examinations of psychological distress and health with older adults in Puerto Rico.

## Limitations

The current study has several limitations. First, our data are from a non-probability sample and are thus not generalizable. However, it remains one of a very limited number of studies that examine older adults living in Puerto Rico during the COVID-19 pandemic. We also used a cross-sectional design and therefore cannot establish directionality between COVID-19 experiences and psychological distress. We recognize that the older adult population in Puerto Rico is heterogeneous in ways that may contribute to psychological distress in minoritized groups (e.g., sexual and gender minorities, Camacho et al., 2022). Our data were collected in 2021 using primarily subjective self-report measures (e.g., experiencing delays in treatment) that may be subject to recall bias. Analyses with more recent data and larger representative samples including vulnerable groups (e.g., individuals with cognitive impairment), and nuanced culturally sensitive-validated objective and subjective measures of potential stressors (e.g., delays in care, outmigration, caregiving burden), moderating factors(e.g., spirituality, intergenerational support, social strain), and mental health (e.g., depression, anxiety, grief) may confirm, qualify or dispute our findings.

### Implications

Future research should examine the longitudinal impacts of COVID-19 experiences on psychological distress in the Puerto Rican context, not least because infections continue to be a public health threat (WHO, 2023). Further, the death toll and disruption of grief processes (Eisma,& Tamminga, 2020), and physical sequelae, including long COVID (Crook et al., 2021; WHO, 2023), underscore a need to explore the impact of COVID-19 experiences on other mental health outcomes in Puerto Rico. Examples include prolonged grief disorder (Gang et al., 2022) and Post-Traumatic Stress Disorder (Kaseda & Levine, 2020). Narrative studies that draw on trauma informed principles will improve understanding of how infected and affected older adults manage chronic stressors, make sense of the pandemic, and cope with COVID-19 stressors and health challenges (Camacho et al., 2023).

Finally, this study provides an important glimpse into the mental health of older adults in Puerto Rico during the COVID-19 pandemic. Given their high proportion in the population, researchers and clinicians should collaborate to identify and implement effective, sustainable mental health interventions and policies for common mental health disorders. Strong bridges with the mainland U.S. can help to develop the nascent Puerto Rican geriatric healthcare infrastructure. Collaborations can also focus on how adapting evidence-based interventions for psychological distress (e.g., Chibanda et al., 2015) to the local context and culture (e.g., Guarnaccia et al., 2003, Negroni et al., 2020). Finally, in addition to professional mental health providers, peer advocates can deliver effective and cost-effective interventions that uplift community strengths already present (Cabassa et al., 2017; Camacho et al., 2018).

## Data Availability

The data that support the findings of this study are available from author, Denise Burnette, upon reasonable request.
